# Coinfection of *Mycoplasma suis* and porcine circovirus type 3 is linked to reproductive failure in pig farms

**DOI:** 10.14202/vetworld.2024.2477-2487

**Published:** 2024-11-07

**Authors:** Tram Ngoc Thi Ngo, Nam Minh Nguyen, Roongroje Thanawongnuwech, Le Minh Thong, Trang Phuong Thi Nguyen, Toan Tat Nguyen, Duy Tien Do

**Affiliations:** 1 Department of Infectious Diseases and Veterinary Public Health, Faculty of Animal Science and Veterinary Medicine, Nong Lam University, HCMC, Vietnam; 2 Center for Genetics and Reproductive Health (CGRH), School of Medicine, National University HCMC, Vietnam; 3 Department of Veterinary Pathology, Faculty of Veterinary Science, Chulalongkorn University, Bangkok, Thailand; 4 Department of Biotechnology, International University, Vietnam National University, HCMC, Vietnam; 5 The Animal Biomedical Research Laboratories, Nong Lam University, HCMC, Vietnam

**Keywords:** coinfection, *Mycoplasma suis*, porcine circovirus type 3, reproductive failure

## Abstract

**Background and Aim:**

Reproductive disorders in swine herds pose significant challenges to pig breeding due to both infectious and non-infectious factors. In large-scale pig farming, coinfections are increasingly common, affecting sow health and herd productivity. This study aimed to determine occurrence and coinfection patterns of *Mycoplasma sui*s and porcine circovirus type 3 in Vietnamese pig farms and to evaluate their association with reproductive disorders and clinical signs in affected herds.

**Materials and Methods:**

We collected 291 samples from 15 farms, composed of whole blood and various tissues from fetuses and weak-born piglets. Molecular biological testing was conducted to detect key pathogens of interest. Consistently, porcine circovirus type 3 (PCV3) and porcine *Hemoplasma* were detected and sequenced for the whole genome and partial *16S rRNA*, respectively. The genetic diversity of PCV3 and *Mycoplasma suis* was analyzed.

**Results:**

Various clinical signs, including abortion, stillborn, mummified, and weak-born piglets, and dermatitis, were recorded. *M. suis* was detected in 252/291 (86.59%) samples from all 15 surveyed farms, with an occurrence of 100%. PCV3 was detected in 35.05% (102/291) samples and 73.3% (11/15) of farms. PCV3 and *M. suis* coinfections were observed in 29.21% of the positive samples. It should be noted that most PCV3 Ct-values were above 30, indicating the existence of PCV3 in the herd but with insufficient data to confirm its pathogenic potential. The complete genomes of 10 PCV3 strains identified in this study exhibited high sequence homology, with >97% nucleotide identity. In addition, the eight partial *16S rRNA* porcine *Hemoplasma* sequences shared absolute identity with *M. suis* isolates from pigs in China and Germany.

**Conclusion:**

This report on the occurrence of *M. suis* and PCV3 in pigs from farms with reproductive failure provides important insights into the expanding global distribution of these pathogens. Our findings warrant further investigations of the pathogenic potential and economic implications of *M. suis* and PCV3 in pigs with reproductive failure in Vietnam.

## Introduction

Reproductive failure in sows is one of the most important factors affecting pig breeding. Many reproductive disorders are linked to both non-infectious and infectious agents. Along with the large-scale and intensive development of modern pig farms, coinfection pathogens are increasingly common in the pig industry [1, 2]. These major pathogens, such as porcine reproductive and respiratory syndrome virus (PRRSV), classical swine fever virus (CSFV), porcine circovirus type 2 (PCV2), porcine pseudorabies virus (PRV), porcine parvovirus (PPV), and Japanese encephalitis virus (JEV), cause reproductive failures in pigs have been reported [3]. PCV3 is considered to be related to reproductive disorder syndrome in pigs [4–7]. Since its first detection in the USA [4, 8], PCV3 has been detected in many countries, suggesting its global distribution [9]. PCV3 can infect pigs of different age groups and production phases and is found in fetuses, nursery pigs, fattening pigs, stillborn, and sows [10, 11]. The virus was found in different tissues, serum, and oral fluids collected from pigs with different health statuses. PCV3 was detected in cases of cardiac and multisystemic inflammation [8]; porcine dermatitis and nephropathy syndrome [8, 12, 13]; respiratory disease [10, 14–16]; congenital tremor in neonatal pigs [5]; periarteritis [13]; reproductive failure, such as abortion, stillbirths, and mummification of fetuses [6, 13, 17–24]; or gastrointestinal signs [10, 16]. On the other hand, many reports have described the detection of PCV3 in pigs without any specific clinical signs [14, 25, 26].

*Mycoplasma suis* is a bacterium whose infection can be significant, especially in breeding herds during the farrowing period [27–31]. *M. suis* was first discovered in 1932 in the United States [32], and the presence of *M. suis* has been recorded in most countries worldwide [33]. *M. suis*, formerly known as *Eperythrozoon suis*, is a bacterium that causes infectious anemia in pigs [31]. Simultaneously, the repercussions of *M. suis* infection can be severe in breeder sows, causing issues such as icterus, hemolytic anemia, fever, and weakness in neonatal pigs, potentially leading to delayed estrus, early embryonic death, and late-gestation abortions [34–36].

The study aimed to determine the occurrence and impact of *M. suis* and PCV) coinfection on reproductive disorders in Vietnamese pig farms, with a focus on clinical manifestations, infection rates, and genetic characteristics of PCV3 and *M. suis* strains.

## Materials and Methods

### Ethical approval

The study was conducted in compliance with the institutional rules for the care and use of laboratory animals and using a protocol approved by the Ministry of Agriculture and Rural Development Vietnam (TCVN 8402:2010).

### Study period and location

The study was conducted from April 2023 to March 2024 through samples collection at farms in Southeast province, Vietnam and analyzed in the Animal Biomedical Research Laboratory (ABR Lab, Nong Lam University – HCMC, Vietnam).

### Sample collection and processing

A total of 291 samples were collected from 15 farms in Vietnam that exhibited clinical signs of reproductive disorders, such as abortion, mummification, stillbirth, weak-born piglets, and dermatitis in sows. These farms varied in production size and type, general hygiene level, and health status. Vaccination programs, including CSFV, Aujeszky’s disease virus (ADV), foot and mouth disease (FMD), PPV, PRRSV, and PCV2 vary by farm. The types of samples included 142 whole blood, 11 colostrum, nine oropharyngeal fluid, eight vulva fluid from sows, and 121 tissue samples (lungs, heart, liver, kidney, spleen, and lymph node) from fetuses (mummified and stillborn) and weak-born piglets. Fresh tissues were homogenized (10% w/v in phosphate-buffered saline) and were maintained at −20°C until use.

### Detection of PCV3, *M. suis*, and other viral pathogens

Viral DNA/RNA from 291 clinical samples was extracted using a commercial kit (GeneJET™ Viral DNA and RNA Purification Kit, Thermo Scientific, Lithuania) according to the manufacturer’s instructions. The DNA/RNA product was converted to cDNA using a RevertAid First Strand cDNA Synthesis kit (Thermo Scientific). PCV3 was detected by real-time polymerase chain reaction (PCR) using the primer PCV3-F: CGCATAAGGGTCGTCTTGGA; PCV3-R: CMGCTCAGCAAACAAAAACTATGT TC and the probe: FAM-TCCAGGCGCCGTCTA GATCTATGGC-BHQ1, as previously described by Yuan *et al*. [37]. These samples were also tested for other pathogens causing reproductive failure, including African swine fever virus (ASFV) [38], CSFV [39], PRRSV [40], PCV2 [41], PRV, PPV, and JEV [42]. Furthermore, *M. suis* was detected by PCR using *M. suis*-F: GCATTGCCCAGTCCCCAAGGA; *M. suis*-R: TGCGGGGAGTACGTGGGAAGG [43].

### PCR amplification, sequencing, and phylogenetic analyses

The PCV3 whole genomes were amplified using two pairs of primers: PCV3-74F: CACCGTGTGAGTGGATATAC; PCV3-1144R: C ACCCCAACGCAATAATTGTA; and PCV3-2F: TTGCACTTGTGTACAATTATTGCG; and PCV3-2R: ATCTTCAGGACACTCGTAGCACCAC [18, 44]. Furthermore, the partial *16S rRNA* gene of porcine hemoplasmas (PH) was sequenced using primer F: HM_16SF1: GAACAGCCGCAATGGGATTGAG and primer R: HM_16SR1: GACCTGGG AACGTATTCACCCTG [33]. Subsequently, PCR products were purified using an ExoSAP-IT^®^ Express PCR product Cleanup Kit (Applied Biosystems^®^, Foster City, CA, USA) according to the manufacturer’s protocol and sequenced using the Sanger method (Macrogen, Korea).

The nucleotide Basic Local Alignment Search Tool (BLAST) (blastn) and protein BLAST (blastp) algorithms available at http://blast.ncbi.nlm.nih.gov/Blast.cgi were used to evaluate the identity of these sequences. The full genome was aligned using ClustalW nucleotides. All reference PCV3 and porcine hemoplasma sequences were obtained from the National Center for Biotechnology Information (NCBI) gene bank (http://www.ncbi.nlm.nih.gov). For phylogenetic analysis, neighbor-joining trees were constructed with the maximum composite likelihood model using the software tool MEGA (version 10.1) (https://www.megasoftware.net/) [45]. To assess the statistical support for each node in the resulting trees, the data were bootstrapped 1000 times using the bootstrap method.

### Statistical analysis

Data analysis was performed using Excel 2010 (Microsoft Office, Washington, USA). One-way analysis of variance was used to compare the occurrence of pathogens. The Chi-square test was used to assess significant differences between groups, and p < 0.05 was considered statistically significant. The binomial and paired-sample t-tests (SPSS Statistics for Windows, v.29.0.2.0; IBM, NY, USA) were performed to compare the positive rate (%) among the different pathogens.

## Results

### Common signs and detection of pathogens in surveyed farms

**Figure-1:**
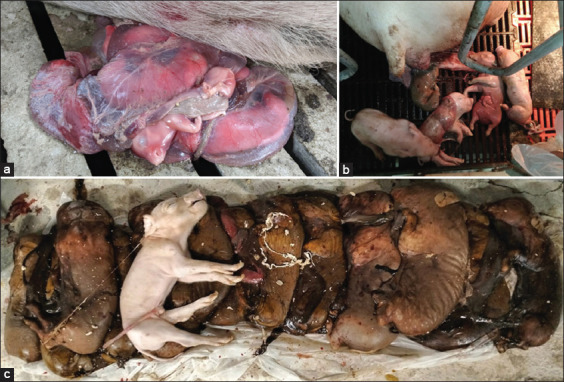
Various clinical signs in different farms included (a) abortions, (b) stillbirths, and (c) mummified.

Table-1 presents the clinical signs observed in pigs from 15 farms. The clinical diversity of reproductive disorders in the surveyed farms included abortion, an increased number of stillbirths per litter and mummification per litter, an increased number of weak-born piglets that died after a few hours, and dermatitis in sows (Figure-1).

**Table-1:** Clinical information was recorded during the week of sample collection.

Farm	Total number of sows	Number of sows surveyed	Clinical sign (%)				Laboratory results (real-time PCR/RT-PCR/PCR)			
	
			Abortion	Mummified	Stillborn	Dermatitis	PCV3	*M. suis*	PCV2	PRRSV
1	2400	/	2.00	5.00	10.0	0.00	+	+	-	-
2	2339	/	0.00	11.0	14.0	0.00	-	+	-	-
3	230	46	0.00	0.80	1.50	0.00	+	+	-	-
4	2477	/	0.00	3.13	6.81	0.00	-	+	-	+
5	2400	/	3.57	-	-	0.00	+	+	+	-
6	2400	300	3.00	2	-	3.33	+	+	-	-
7	596	/	0.00	2.54	5.04	0.00	-	+	-	+
8	1800	/	1.65	2.13	3.98	0.00	+	+	-	-
9	1269	/	1.48	1.45	3.27	0.00	+	+	-	-
10	1320	/	0.00	3.7	5.42	0.00	+	+	-	-
11	1650	/	1.97	3.12	4.68	0.00	+	+	+	-
12	1350	/	0.00	5.40	9.50	0.00	-	+	-	+
13	2269	100	0.00	26.57	22.31	0.00	+	+	-	-
14*	2400	/	7.7	/	/	0.00	+	+	-	-
15*	2400	/	7.82	1.80	3.02	0.00	+	+	-	-
Tổng	27300	/	/	/	/	/	11/15	15/15	2/15	3/15

* Abortion at 10–14 weeks of gestation. “/”=Not applicable, *M. suis=Mycoplasma suis*, PCV3=Porcine circovirus 3, PRRSV=Porcine reproductive and respiratory syndrome virus, RT-PCR=Reverse transcription polymerase chain reaction, PCR=Polymerase chain reaction

### Occurrence of reproductive disorders pathogens in farms

**Figure-2:**
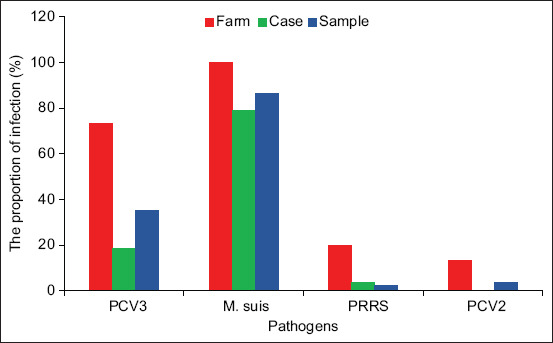
Detection rates of pathogens at farm, case, and sample levels.

PCV3 was found in 11/15 (73.33%) of the 15 surveyed farms. At the case and sample levels, 16/86 cases (18.6%) and 102/291 samples (35.05%) tested positive for PCV3, respectively. *M. suis* was detected in 252/291 (86.59%) samples from all 15 surveyed farms, with an occurrence of 100%. At the case level, *M. suis* was found in 68/86 cases (79.07%). In addition, PCV2 was detected in samples from 2/15 farms (3.78%), whereas PRRSV was detected in samples from 3/15 farms accounting for 7/291 samples (2.41%). Notably, no other pathogens associated with reproductive disorders (ASFV, CSFV, PPV, PRV, and JEV) were detected (Figure-2).

**Figure-3:**
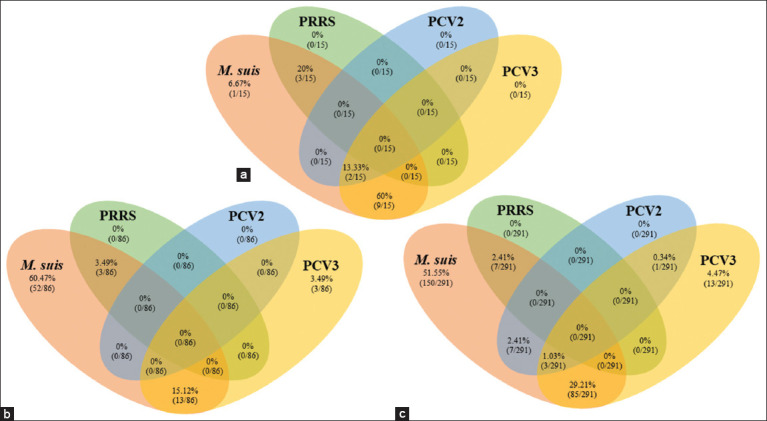
Single infection and coinfection among pathogens at (a) farm level, (b) case level, and (c) sample levels. A case is determined positive when the whole blood of the sow and the aborted fetus or weak-born piglet are both positive for the same pathogen.

Coinfection of PCV3 with other pathogens has been reported by Chen *et al*. [46]. Therefore, we also examined whether PCV3 was concurrent with other major pathogens, such as ASFV, CSFV, PRRSV, PCV2, PPV, PRV, JEV, and *M. suis*. Among the 291 clinical samples, 102/291 (35.05%) were PCV3-positive, 13/102 (12.75%) were single infections, and 89/102 (87.25%) were mixed infections with other pathogens. In addition, *M. suis* was detected in the surveyed farms, with a positive result of 252/291 (86.59%). Among these *M. suis*-positive samples, 150/252 (59.52%) were single infections and 40.48% were coinfected at farms (Figure-3). The highest positive rate of mixed infections was observed in cases in which both PCV3 and *M. suis* were present 85/291 (29.21%), followed by *M. suis* co-infected with either PCV2 or PRRSV 7/291 (2.41%). Triple infection was also observed, with 3/291 (1.03%) cases involving PCV3, PCV2, and *M. suis*.

### Detection rate of pathogens in different sample types

The single positive rate of PCV3 in pigs across sample types ranged from 2.1% to 27.3%, including 3/142 (2.1%) in blood from sows, 3/11 (27.3%) in colostrum, 1/9 (11.1%) in oropharyngeal fluid, 5/32 (15.6%) in mummified samples, and 1/46 (2.2%) in stillborn. However, the occurrence of M. suis single infection ranged from 43.8% to 75% in different samples, including 70/142 (49.3%) in whole blood of sows, 6/11 (54.5%) in colostrum, 6/9 (66.7%) in oropharyngeal fluid, 6/8 (75%) in vulva fluid, 14/32 (43.8%) in mummified samples, 29/46 (63%) in stillborn, and 19/43 (44.2%) in weak-born piglets. In addition, positivity for PCV3 in weak-born piglets was highest at 24/43 (55.81%), followed by whole blood of sows at 50/142 (35.21%), mummified tissue at 9/32 (28.13%), and stillborn at 9/46 (19.57%). The difference in infection rates among these samples was statistically significant (p < 0.01). PCV3 infection rates were higher in the whole blood of sows compared to stillborn (p < 0.05) and higher in weak-born piglets than in whole blood of sows (p < 0.05).

The rate of coinfection between PCV3 and *M. suis* was 51.2% (95% CI: 35.5%–66.7%) in weak-born piglets compared with 31.7% (95% CI: 24.1%–40%). The positive rates of colostrum, oropharyngeal fluid, vulva fluid, stillborn, and mummification tissues displayed positive rates of 18.2% (95% CI: 2.3%–51.8%), 22.2% (95% CI: 2.8%–60%), 25% (95% CI: 3.2%–65.1%), 17.4% (95% CI: 7.8%–31.4%), and 12.5% (95% CI: 3.5%–29%), respectively. The coinfection of pathogens among different sample types is presented in Table-2.

**Table-2:** Percentage of positive samples (% and CI) from various samples.

Samples	Single infection (%)		Co-infection (%)				
	
	PCV3	*M. suis*	PCV3+PCV2	PCV3+*M. suis*	PCV2+*M. suis*	*M. suis*+PRRSV	PCV3+PCV2+*M. suis*
Whole blood (N=142)	2.1 (CI: 0.4–6.0) (3/142)	49.3 (CI: 40.8–57.8) (70/142)	0.7 (CI: 0-3.9) (1/142)	31.7 (CI: 24.1–40.0) (45/142)	4.2 (CI: 1.6–9.0) (6/142)	2.8 (CI: 0.8–7.1) (4/142)	0.7 (CI: 0–3.9) (1/142)
Colostrum (N=11)	27.3 (CI: 6.0–61.0) (3/11)	54.5 (CI: 23.4–83.3) (6/11)	/	18.2 (CI: 2.3–51.8) (2/11)	/	/	/
Oropharyngeal fluid (N=9)	11.1 (CI: 0.3–48.2) (1/9)	66.7 (CI: 29.9–92.5) (6/9)	/	22.2 (CI: 2.8–60.0) (2/9)	/	/	/
Vulva fluid (N=8)	/	75.0 (CI: 34.9–96.8) (6/8)	/	25.0 (CI: 3.2–65.1) (2/8)	/	/	/
Stillborn tissue (N=46)	2.2 (CI: 0.1–11.5) (1/46)	63.0 (CI: 47.5–76.8) (29/46)	/	17.4 (CI: 7.8–31.4) (8/46)	/	6.5 (CI: 1.4–17.9) (3/46)	/
Mummified tissue (N=32)	15.6 (CI: 5.3–32.8) (5/32)	43.8 (CI: 26.4–62.3) (14/32)	/	12.5 (CI: 3.5–29.0) (4/32)	3.1 (CI: 0.1–16.2) (1/32)	/	/
Weak-born piglet tissue (N=43)	/	44.2 (CI: 29.1–60.1) (19/43)	/	51.2 (CI: 35.5–66.7) (22/43)	/	/	4.7 (CI: 0.6–15.8) (2/43)

CI, 95%=Confidence interval for the sample positivity rate, “/”=Not applicable, *M. suis*=*Mycoplasma suis*, PCV3=Porcine circovirus 3, PRRSV=Porcine reproductive and respiratory syndrome virus

### PCV3 loads in various samples

**Figure-4:**
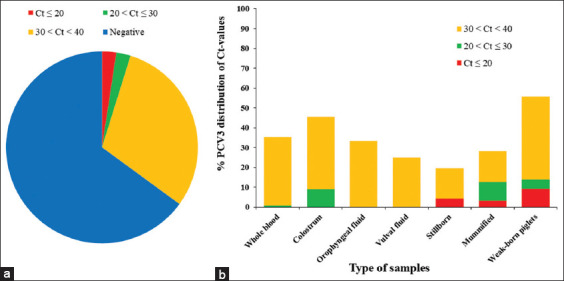
Distribution of crossing threshold (Ct) values for (a) porcine circovirus 3 (PCV3)-positive samples and (b) proportion of PCV3-positive samples according to Ct values for different sample types.

PCV3 DNA was detected in 102/291 samples (35.05%) and 16/86 cases (18.60%). Among these samples, PCV3 load (Ct-values) was <20, indicating potential clinical significance, in 7/291 samples, constituting 2.41% of the total. The overall proportion of PCV3-positive samples was notably high (88/291, 30.2%) (Figure-4a).

PCV3 load by real-time PCR was divided into three gradients, including Ct-value ≤20, 20 <Ct-value ≤30, 30 < Ct-value < 40, and the proportion of samples within each gradient was calculated. More than 9% of the positive samples of PCV3 in the colostrum and mummified tissue had 20 < Ct-value ≤ 30, and most were in the range of 30 < Ct-value < 40. Notably, Ct-value ≤20 of the positive samples in the stillborn, mummified, and weak-born piglets were 4.35%, 3.13%, and 9.3%, respectively (Figure-4b).

### Phylogenetic analysis of the PCV3 complete genome

To better understand the phylogenetic relationships of PCV3 identified in this study, a phylogenetic tree was constructed using the whole-genome sequence of PCV3. In total, 30 complete genome sequences available in the NCBI database were compared with 10 complete genome sequences from this study. The complete genome sequences of the current PCV3 strains were deposited in GenBank (NCBI) under accession numbers OR059205 and OR738334-OR738342.

**Figure-5:**
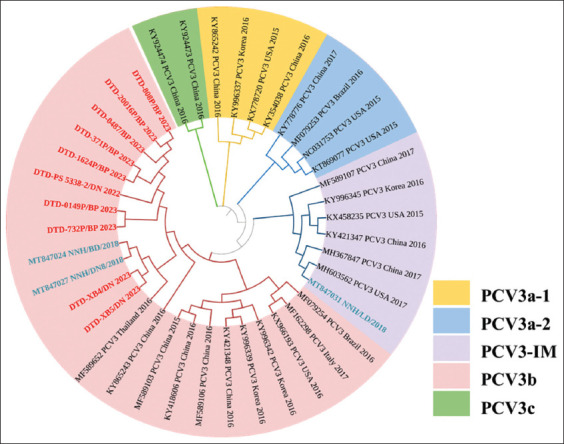
Phylogenetic analysis of porcine circovirus 3 based on complete coding sequences. The tree was constructed using MEGA version XI with 1000 bootstrap replicates. The 10 sequences introduced in this study are indicated in red, and the three reference sequences are indicated in blue from previous research in Vietnam.

The results of this study are consistent with a previous study by Fu *et al*. [47], showing that PCV3 strains are divided into three distinct clades: PCV3a, PCV3b, and PCV3c. The PCV3 strain identified in this study belonged to the PCV3b clade. The phylogenetic tree shows that the current PCV3 strain is closely related to the Vietnam and South China isolates (Figure-5).

### Phylogenetic analysis of *M. suis* partial 16S rRNA

**Figure-6:**
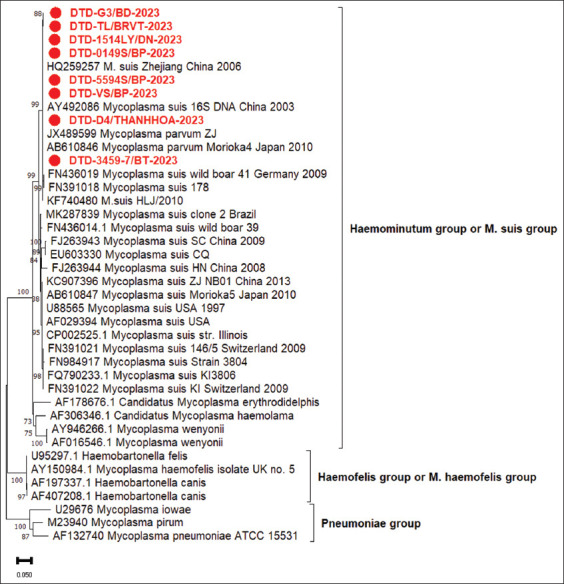
Phylogenetic relationships and proportions of character changes within *Hemoplasma* species based on an approximate 1000-bp partial sequence alignment of the *16S rRNA* gene.

To confirm the PCR results and evaluate sequence variations among *M. suis* strains from different farms in Vietnam, the partial *16S rRNA* gene of eight strains was sequenced. *The M. suis* strains in this study revealed 99.7%–100% identity with their genomes. To establish relationships between *M. suis* isolates and other *Hemoplasma* species, the *16S rRNA* sequences of *M*. *suis* were aligned with previously published sequences from GenBank. The phylogenetic tree was constructed using neighbor-joining in MEGA X (https://www.megasoftware.net/). All *Hemoplasma* species were located within a single clade in the *16S rRNA* gene phylogenetic tree. The *Hemoplasma* species were subdivided into two distinct groups, one containing *Mycoplasma wenyoni*, *M. suis, Mycoplasma parvum*, and *Candidatus Mycoplasma haemominutum* and the other containing *Mycoplasma haemofelis* and *Mycoplasma haemocanis*. The phylogeny showed that all *Hemoplasma* species were most closely related to the *Mycoplasma pneumoniae* group (Figure-6). In the subcluster of *M. suis*, all *M. suis* strains in this study were most closely related to *M. suis* (HQ259257, AY492086, FN436019, FN391018, KF740480). The eight *M. suis 16S rRNA* sequences shared high levels of identity with those from pigs in China (99.5%–99.9%; strain AY492086, KF740480, HQ259257), and Germany (99.2%–99.5%, strain FN391018, FN436019), respectively. The partial *16S rRNA* genome sequences of the current *M. suis* strains have been deposited in GenBank (accession number pending).

## Discussion

PCV3 and *M. suis* were found at the highest rates in this study, while ASFV, CSFV, PPV, PRV, and JEV were not detected. PRRSV and PCV2 were detected in <10% of these farms. It should be noted that scientific and clinical reporting data on the prevalence of *M. suis* are often limited but the significance of *M. suis* prevalence should not be ignored. This study showed that the *M. suis* detection rate was high in sows (86.59%), similar to other studies, at 70.90%–80% [48–50]. However, a Swedish study found *M. suis* infection rate of only 19% [51]. Therefore, the difference in the occurrence of *M. suis* could be due to the chronic infection stage, which is difficult to detect. In this study, 11/15 farms (73.33%) and 102/291 samples (35.05%) were positive for PCV3. The occurrence of this virus at the herd and sample levels was 73.33% and 35.05%, respectively, higher than previously reported by Zheng *et al*. [25] and Kwon *et al*. [52]. Higher PCV3 prevalence has been reported in some countries, with detection at 24/35 farms (69%) in China [18], 53/73 farms (73%) in UK [53], and 12/14 farms (86%) in Poland [55].

PCV3 was detected in pigs with various clinical signs related to reproductive disorders. However, confirming the association between the virus and any particular clinical signs or lesions is challenging. There were no significant associations between specific clinical signs and the presence of PCV3. Unfortunately, this study has some limitations: (1) Various pathogens and coinfections can cause similar clinical signs, and (2) clinical information could not be obtained sufficiently because of the restricted access and entry to the farm required to ensure biosecurity.

PCV3 in fetuses, weak-born piglets, and sows suggests that the virus can induce reproductive problems. Numerous investigations have revealed that PCV3 is present in a wide range of specimens, such as fetal thoracic fluid, serum, heart, lung, brain, lymph node, liver, kidney, spleen, tonsil, peritoneal cavity, intestine, colostrum, umbilical cords, and salivary glands [4, 5, 13, 14, 21, 49, 25, 52–54]. Factors potentially influencing the prevalence of PCV3 include biosafety, biosecurity, management of pig flow, environmental conditions, and individual factors, such as existing herd immunity, the presence of other microorganisms, and infection by immunosuppressive pathogens [55]. Although it cannot be proven that PCV3 found herein was the cause of reproductive losses in the studied farms, coinfections with other bacterial pathogens might account for reproductive disorders [26, 56, 57]. In this study, severe clinical signs were associated with *M. suis* infection in sows, including acute and chronic anemia, pyrexia, anorexia, hypoglycemia, icterus, and reproductive disorders [30, 36]. Comparable studies on the detection rate of *M. suis* in various studies on reproductive disorders are rare, and its potential to cause reproductive disorders has not been proven. The high numbers of stillborn and mummified fetuses on *M. suis*-positive farms must be interpreted with caution because other pathogens can induce similar reproductive failure. However, *M. suis* was detected in most of the studied farms, suggesting that this pathogen is widespread in Vietnamese pig herds. However, its pathogenic role in sows and fetuses needs to be clarified. Notably, PCV3 and *M. suis* coinfection was detected at a high rate (30.67%). Note that the widespread use of effective vaccines could explain the lack of detection of CSFV, PPV, and PRV in this study.

The viral load of PCV3 (Ct-value) distribution was analyzed to evaluate its pathogenicity. Cases with a high Ct value show a high viral load, which may be significant in some cases, depending on the microscopic lesions, clinical history, and stage of infection. The consideration of clinical and pathological contexts is important in the interpretation of PCV3 molecular testing results. The average Ct values of these positive samples were >30, indicating low viral loads in whole blood, fluids, stillborn, and weak-born piglets. However, the mummified tissues comprised nine positive samples whose Ct values ranged from 14.27 to 36.67, with an average value of 28.46. These findings suggest that mummified tissue had a higher detection rate and load than other samples.

The full-length genomic sequence of the PCV3 isolate was determined using metagenomics sequencing. Phylogenetic analysis has been widely used to characterize PCV3 sequences in clinical samples from multiple countries [26]. These strains shared 97.25%–100% nucleotide similarities with the available PCV3 reference strains from NCBI GenBank. This implies that PCV3 has high genetic stability, which is concordant with findings from a previous study by Qi *et al*. [58]. Furthermore, the PCV3 strains identified in reproductive failure cases in this study were classified as subtype 3b. Nguyen *et al*. [59] reported the presence of the PCV3a genotype in pig herds in the Central and Southern regions. The whole genome of PCV3 was analyzed, and the PCV3b strain was first reported by Nguyen *et al*. [60] in Vietnam in 2021. Dinh *et al*. [61] sequenced the ORF2 segment of 29 PCV3 strains; 23/29 strains belonged to the PCV3b genotype, 5/29 belonged to the PCV3a genotype, and for the first time, PCV3c was reported (1/29). Therefore, PCV3 has high diversity (PCV3a, PCV3b, and PCV3c) circulating in Vietnamese pig herds, in which PCV3b is the most predominant genotype.

Due to the high identity between the sequences of *M. suis* and *M. parvum*, which were identified as the same groups in the phylogenetic analysis, the detection of *Mycoplasmas* by both qPCR and PCR techniques [62] evidenced the need for later sequencing and phylogenetic analysis to differentiate between PH. Based on the sequences of the *16S rRNA* gene of *Mycoplasmas*, the phylogenetic tree formed two distinct clusters to determine the presence of porcine *Hemoplasmas* in the reproductive disorders farm system in Vietnam. Porcine *Hemoplasma* was present in all surveyed farms, and its identity relative to the reference *M. suis* strains ranged from 95.05% to 99.90%. In addition, studies on circulating porcine *Hemoplasmas* using *16S rRNA* have been published in several countries [31, 48]. However, the pathogenic role of these species in the pig industry is unknown. Therefore, further research on epidemiology is needed to identify possible reservoirs, transmission routes, and pathogenesis of porcine *Hemoplasmas* to control its spread. In-depth studies on the genetic diversity of porcine *Hemoplasma* are needed to understand their pathogenic potential.

Recent regulations in Vietnam, such as Decree No. 13/2020/ND-CP and Decree No. 46/2022/ND-CP, no longer allow preventive antibiotics in adult pigs, pregnant sows, and lactating sows. Therefore, many sow farms and farms in this survey stopped using antibiotics that were routinely mixed into feed to prevent disease. However, antibiotic treatment was a herd protection factor that controlled *M. suis* infection [63]. This may be a risk factor for the re-emergence of *M. suis* infection. During pregnancy, sows may be susceptible to bacterial and viral pathogens, especially respiratory diseases and *M. suis* infection. Several drugs have been reported to effectively treat *Eperythrozoon* (previous name of *M. suis*) infections, including doxycycline, oxytetracycline, tetracycline, chlortetracycline, and imidocarb dipropionate [64–66]. However, the prevention and treatment of *M*. *suis* infection using oxytetracycline are controversial. Oxytetracycline can be used for acute outbreaks or to prevent signs and secondary anemia before the stress period [67]. This treatment can often control clinical signs. Supportive treatment and iron dextran injection may also be indicated for rapid recovery and reduced mortality [68]. Reducing the use of antibiotics to avoid the risk of drug resistance is a correct and necessary strategy; however, alternative solutions to antibiotics or preventive therapy for infectious bacterial pathogens, such as *M. suis*, need to be considered seriously, such as vaccine development.

## Conclusion

This study highlights the need for specific attention to *M. suis*, its high occurrence in reproductive disorder farms, and the significant coinfection rates with PCV3. This is the first report on the occurrence of these pathogens in Vietnam, raising questions about their combined impact and pathogenesis on swine reproductive health and emphasizing the importance of further research in this critical area.

## Data Availability

The data that support the findings of this study are available from the corresponding author on a reasonable request.

## Author’s Contributions

DTD and NMN: Designed the study. TNTN, NMN, TPTN, and DTD: Performed experiments. TNTN, RT, LMT, and TTN: Analyzed the data. DTD, RT, NMN, and TNTN: Drafted and revised the manuscript. All authors have read and approved the final manuscript.
